# Does social trust slow down or speed up the transmission of COVID-19?

**DOI:** 10.1371/journal.pone.0244273

**Published:** 2020-12-17

**Authors:** Jungwon Min

**Affiliations:** Faculty of Economics and Management, Sophia University, Tokyo, Japan; Middlesex University, UNITED KINGDOM

## Abstract

Social trust has been an important mechanism in overcoming crises throughout history. Several societies are now emphasizing its role in combating the COVID-19 pandemic. This study aims to investigate how variations in social trust across 68 countries are related to the transmission speed of COVID-19. Specifically, using cross-national index data from the World Value Survey, the study tests how variations in social trust across countries generate different time durations at which each country reaches the peak in terms of increases in new infections of COVID-19. Using data drawn between December 31, 2019 and July 31, 2020, this study found that in countries with a high level of social trust, particularly trust among ingroup members, or with a narrower or wider range than the intermediate range of trustees, the number of new infections tended to reach the first peak within a shorter time duration than in other countries. These results imply that in such societies, on the one hand, high cooperation among people to achieve common goals and strong compliance to social norms may allow them to begin neutralizing COVID-19 faster. On the other hand, however, the low risk perception and prevalence of cohesive relationships among people may lead to speedier transmission of COVID-19 before neutralization takes place.

## Introduction

Trust is widely considered an important type of social capital [1, 2]. A number of previous studies based on social capital theory have proved that social trust is a substantial factor in facilitating socio-political cooperation among social actors and that it also generates several benefits [1, 3]. For instance, individuals who express a high level of social trust are more likely to be physically and psychologically heathier than others [4, 5]. Organizations embedded in a high-trust society or trustworthy transaction relationships exhibit higher CSR performance [6] and economic performance [7]. Social communities with a high level of trust have low crime rates [8]. Countries with strong trust among their citizens tend to enjoy economic development [9] and democratic stability [10].

Owing to these benefits, throughout history, societies have often encouraged people to trust each other and be trustworthy when faced with global adversities such as economic or institutional crises [11]. The COVID-19 pandemic does not present a different situation. According to various governments, mass media, and scholars, social trust will play a key role in mitigating the spread of COVID-19 (e.g., see Schrad’s article [12]). Following this, some articles, based on case studies of countries such as South Korea and Singapore, have noted that social trust in their public sectors have significantly contributed to the control and slowdown of the spread of COVID-19 [13].

Will societies with strong social trust among people decelerate the transmission of COVID-19? Although some high-trust societies may have successfully curbed the spread of COVID-19, it is still too early to conclude that social trust will control the spread COVID-19 for three reasons. First, to date, there is little empirical evidence of the significant relationship between the degree of social trust and epidemics. Second, as prior observations have mainly focused on trust in local governments (e.g., see study by Kye & Hwang [13]), it still remains unknown whether and how social trust among general people can affect the spread of COVID-19. Third, the COVID-19 pandemic is distinct from other crises that we have encountered in history in that it worsens with direct social interaction. Social capital theory argues that social trust forms through cohesive relationships among actors based on their frequent and intimate direct interactions [1, 3, 14]. In this regard, social distance in high-trust societies represents a somewhat paradoxical requirement that contradicts the mechanism (i.e., social trust) that has sustained societies.

This study explores the relationship between social trust and the transmission speed of COVID-19 worldwide. Using cross-national index data from the World Value Survey (WVS) 2017–2020 [15], the study examines how variations in social trust across countries create different time durations at which each country reaches the first peak in terms of increases in new infections of COVID-19 (“the fast peaking time”). This empirical challenge has critical implications for both practice and theory. Although epidemics have been among the world’s significant social issues, we still have scant empirical evidence regarding their antecedents from a socio-behavioral perspective. In the current absence of pharmaceutical interventions, however, the most effective mitigating response to the pandemic is the change of behavior concerning prosocial health promotion that should be imposed on people. For this reason, Van Bavel et al. [16] mentioned that the research in the discipline of social and behavioral sciences has an important role in providing valuable insight for the management of this pandemic. Social trust is such a key construct that controls human behaviors because it can define social norms and compels people to stay compliant to those norms [17]. Therefore, the results of this study have implications for controlling further escalations of COVID-19 and other similar crises in the future from a socio-behavioral perspective.

In addition, given that social distance contradicts social trust at a certain level, an emphasis on social distance in high-trust societies generates an unexpected paradox for the social actors involved. The results will show how social actors behave under this paradox in high-trust societies and extend the literature on social trust.

The rest of this paper is organized as follows. The second section introduces the concept and types of social trust based on the previous literature and predicts how social trust influences the speed of the spread of COVID-19. The third section describes the methods used to test the predictions, and the fourth section reports the results. The final section discusses the implications of the findings for both practice and the literature.

## Background and hypothesis

### Definition and types of social trust

Social trust has been defined as the belief that other people will not cause any harm to us but will rather look after our interests [18]. It is a psychological state comprising the intention to accept vulnerability based on positive expectations of others [19] and the expectancy that a message received is true and reliable [20]. Despite these diverse definitions, the literature commonly emphasizes that in societies with a high level of social trust, people have an institutionalized expectation that others will behave reciprocally for their common benefit and will accept vulnerability from each other.

Social trust is based on interactions between the actor placing the trust, namely, the trustor, and the target, namely, the trustee [21]. Previous studies have introduced two types of social trust based on these two components: particular and general trust [14, 22]. Particular trust refers to the degree to which people trust their ingroup members with whom they interact closely and frequently, such as family members, friends, colleagues, and neighbors. This type of trust is formed based on interpersonal networks and knowledge gained through historical face-to-face communication. In contrast, general trust indicates the level of trust in unfamiliar members of society, namely, outgroup people. In a society with a high level of general trust, people tend to strongly believe that even strangers are reliable and honest and that they act favorably [21, 23]. By this definition, some scholars have seen general trust as the only true form of trust based on unconditional human benevolence [23], arguing that it is distinguished from the particular trust formed through certain kinships or conditional interests. The literature on social trust has considered these two types of trust as major sources of social capital and has examined their determinants such as economic equality, homogeneity in ethnic or religious factors, and democracy [18, 24].

Previous studies have also suggested another concept that determines trust in society: the trust radius, which is defined as a circle of people among whom trust is operative [25]. This concept refers to the breadth of trustees, which is distinct from social trust, which indicates the level or degree of trust among ingroup or outgroup trustees. Delhey and his colleagues [25] treated the trust radius as independent of the two types of social trust but viewed the actual amount of trust in a society as depending on both social trust and the trust radius. When people have a narrow range of trustees, the reliance on ingroup people will increase, which may lead to more cohesive relationships within the group. In contrast, when people have a wide range of trustees, they will harbor trust toward various members of society. Such a society will become more grounded based on trust and motivate people to accept vulnerability from others. Adopting this perspective, this study also considers that the influences of social trust will rely not only on its levels but also on its radius.

### Influence of social trust on the spread of COVID-19

This study investigated how the different levels and width of social trust in each country affect the fast peaking time for COVID-19, measured by the time taken for the number of new COVID-19 infections to reach the first peak. Here, fast peaking has two different meanings. First, it refers to the steep slope of increase in the new infections before the peak—that is, the transmission speed of COVID-19 is fast after the country finds its first case. Second, it refers to the faster control of such transmission because the peak indicates a starting point for the decrease in the number of new infections. However, fast peaking does not measure a steep or flat slope after the peak to approach the normal state. The neutralization speed of COVID-19 is less testable at the current moment because COVID-19 is still in progress.

In countries with high levels of particular and general social trust, the number of new infections will reach the peak faster. In other words, these countries will experience faster tramsmission and neutralization of COVID-19. The following two reasons support faster transmissions. First, social trust is not created in a single moment but rather builds up gradually through historical interactions among people [26]. Therefore, in countries with high levels of particular and general trust, several aspects of traditions and social systems become rooted in frequent and deep face-to-face interactions among people within their ingroup or the entire society. As such routines reflect the cultural dimension of the countries, they also represent long-lasting national characteristics [27]. This rooted nature of social trust may make it challenging for people in high-trust countries to abide by the urgent demand for social distancing. Far from maintaining social distance, the precarious situations may promote more cohesive interactions among people to overcome common adversities, and this may persist until they perceive its high risks.

Second, previous studies have shown that people in high-trust societies tend to take high risks. For instance, Siegrist et al. [28] used survey data from Switzerland and showed that people with a high level of trust in authorities, managers, and/or scientists have low risk perception regarding technological, societal, and natural hazards. Similarly, Su et al. [29], using the Chinese context, proved that in regions with high social trust, organizational decision-makers are willing to accept high risks and approve risky proposals. According to these studies, people in high-trust environments accept unfamiliar situations easily and are less afraid of getting into trouble. This is because they have a confident perception that others who are connected with them or their authorities in society will help them when they face problems. These positive expectations and sense of security may be reflected in health emergencies, such as the COVID-19 pandemic.

On the other hand, in high-trust societies, once people recognize the risks in society, they will immediately control their behaviors that may amplify such risks further. The two following reasons support this idea. First, high-trust societies are characterized by cohesive relationships among people. In these societies, people tend to be oriented toward a common goal and perceive strong responsibility to contribute to that goal [17]. This orientation will be critical to mitigate the pandamic because fighting it requires large-scale and long-term cooperation among people [16]. Without an orientation toward a collective goal, people may pursue self-convenience and be less likely to take prosocial behaviors that protect the health of other members in the society.

Second, once a common goal is established in high-trust societies, compliance to social rules and norms should follow suit as a medium of achieving the common goal. Several previous studies have pointed out the importance of compliance to social rules in managing COVID-19 transmissions. For instance, scholars have shown that the higher infection ratios and morality of males, in comparison to females, are relevant regarding gender differences in compliance to health rules imposed by societies. That is, females are more risk averse, concerned about health consequences, and in favor of activity-restraining public policy measures than males. These differences allow females to be more compliant with the social rules for health-promoting behaviors during the COVID-19 pandemic such as washing one’s hands, maintaining distance from others, and staying at home [30, 31]. Social capital theory points out that in high-trust societies, compliance to rules tend to be strong, and any deviations tend to be sanctioned promptly [1, 25]. In such societies, people are more likely to be concerned about common, rather than their own, benefits [32]. Hence, after people perceive the common risk from the rapid increase in the number of new infections, they will react promptly to control the situation by taking prosocial behaviors to promote common health, so neutralization will follow quickly.

Finally, as assumed, if both particular and general trust accelerate the fast peaking time for the number of new infections, the trust radius will form a U-shaped relationship with the acceleration. In countries with a narrower range of trustees, people have intimate interactions within ingroups and rely heavily on ingroup members. This can facilitate both frequent contact among the ingroup members and sensitive reactions to risks posed to them. Where there is a wider range of trustees, people are generally embedded in high trust-based environments. In these societies, people are likely to have cohesive forms of social contact, low risk perception, and sensitive reactions to the common risks for society. The intermediate range of trustees cannot take either of these effects, so the fast peaking time will be less likely in countries with an intermediate trust radius.

Following these arguments, this study predicts that (1) in countries with a high level of particular or general social trust, the number of new infections of COVID-19 reaches its peak faster, and (2) the relationship between the trust radius and the fast peaking time for the number of new infections is U-shaped. Specifically, in countries with a narrower or wider trust radius rather than an intermediate one, the number of new COVID-19 infections reaches its peak faster. The analysis results provide general support for these predictions and show a significant relationship between social trust in the countries and the fast peaking time for COVID-19.

## Materials and methods

### Data

To test the influence of social trust, this study used data from the World Values Survey (WVS), which is the largest cross-national survey investigating different attitudes, values, and beliefs across countries and has been carried out since 1981. The most recent investigation is the WVS 7th wave, which took place between 2017 and 2020, in which about 104,000 respondents across 80 countries participated [15]. This survey comprised several sections such as “social values, attitudes, and stereotypes,” “happiness and well-being,” “social capital, trust, and organizational memberships,” “corruption,” and so forth [15]. Following previous studies on social trust [14, 25, 33], this study focuses on questions and answers in the “social capital, trust, and organizational memberships” section in the WVS 7th wave [15].

The data on COVID-19 infections around the world were collected from the European Centre for Disease Prevention and Control (ECDC) [34]. These data include information on COVID-19 infections in 211 countries since December 31, 2019 [34]. The final dataset combined with the WVS data covered 68 countries (4 from Africa, 20 from Asia, 31 from Europe, 4 from North America, 2 from Oceania, and 7 from South America) after excluding missing values [15, 34].

### Analysis model

The dependent variable in this study is the time duration each country takes to reach the peak of new infections. This study uses the number of new infections per million per day in each country as its measure. This measure is more effective than the use of the direct number of new infections in order to control the inherent differences in population sizes across countries. To estimate the association between social trust and the fast peaking time of new infections, this study applied the Cox proportional hazards model, following other similar studies such as those on the outbreak of influenza [35]. This model is more appropriate than other parametric event history models given that it does not assume any baseline hazard ratio for the events. The hazard rate is calculated using [36]:

$${\text{h}}_{\text{i}}(\text{t})={\text{h}}_{0}(\text{t})\text{exp}(\beta_{1}{\text{X}}_{\text{i}1}+\beta_{2}{\text{X}}_{\text{i}2}+\ldots +\beta_{\text{k}}{\text{X}}_{\text{ik}})$$

where h_0_(t) is the baseline hazard function, and β_1_ to β_k_ are the vectors of parameters for k explanatory variables. According to this formula, the different hazard rates across countries rely on the explanatory variables and not on the baseline hazard. The sample period commenced on December 31, 2019 and ended on July 31, 2020. Each country was at risk when it had COVID-19 infections for the first time. The dataset entered the number of new infections every day for sample countries since the countries acquired the first case until either the number reached its peak or the end of the study period.

To define the peak, this study developed a dummy variable coded as 1 when the given day met two conditions and otherwise coded as 0. The two conditions are as follows. First, the given day has the largest number of new infections between 14 days prior to and 14 days after that day. The 14 day-interval is appropriate because the maximum incubation period for the disease is 14 days. People would need around 14 days to recognize the risk of the spread of COVID-19. Second, the cumulative number of cases on that day should be larger than one for every million. With the application of the second condition, the peak cannot be determined until each country reaches a certain level of total cases.

In the sample periods, some countries experienced multiple peaks by experiencing multiple cycles of drastic increases and decreases in new infections after they neutralized the first wave successfully. As these additional waves may have arisen from more complicated sources across countries, this study only focuses on the first peak and excludes data after it.

### Explanatory variables

**Fig 1:**
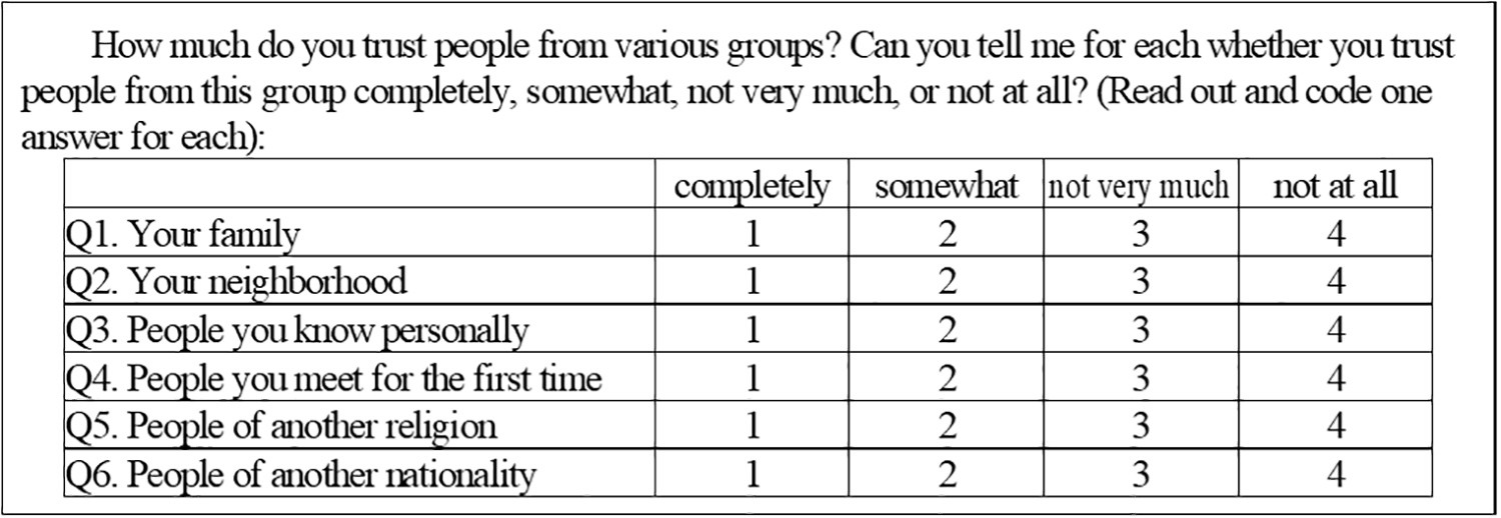
WVS questionnaire.

The first explanatory variable in this study comprises both types of social trust, namely, particular and general, in each country. Previous studies have defined these trust levels based on the following questions (as shown in Fig 1) in the WVS [14, 15, 25]:

The first three questions determine the trust levels for the ingroup, namely, particular social trust, whereas the last three concern those of the outgroup, namely, general social trust. To make the larger values represent higher levels of trust, each answer was rescaled to 0 (no trust), 0.33, 0.66, and 1 (completely trust). Then, the averages of the rescaled answers from Q1 to Q3 and those from Q4 to Q6 per country were calculated, and each was defined as *particular* and *general trust* of the country, respectively.

**Fig 2:**
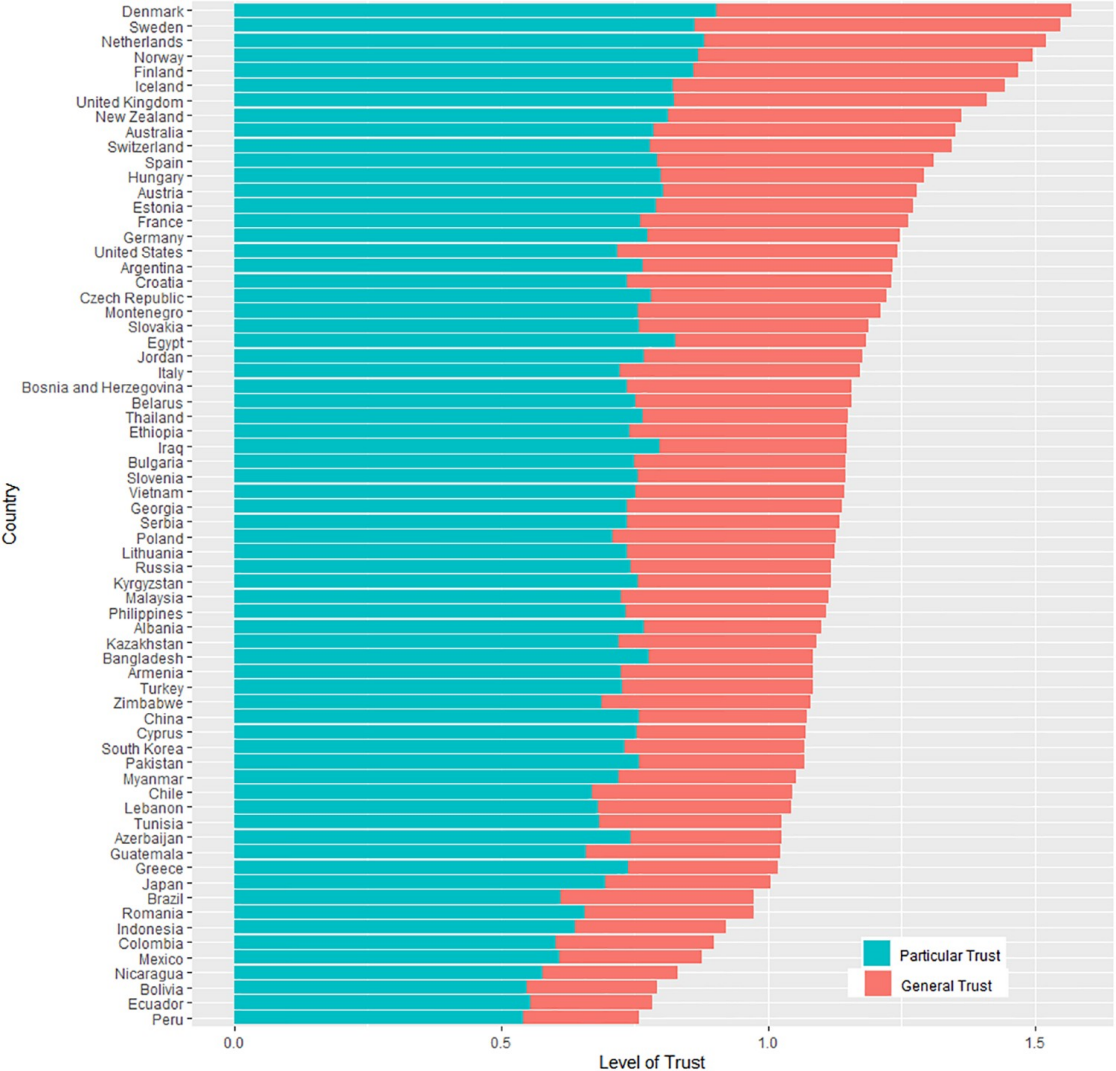
Social trust across countries.

**Fig 3:**
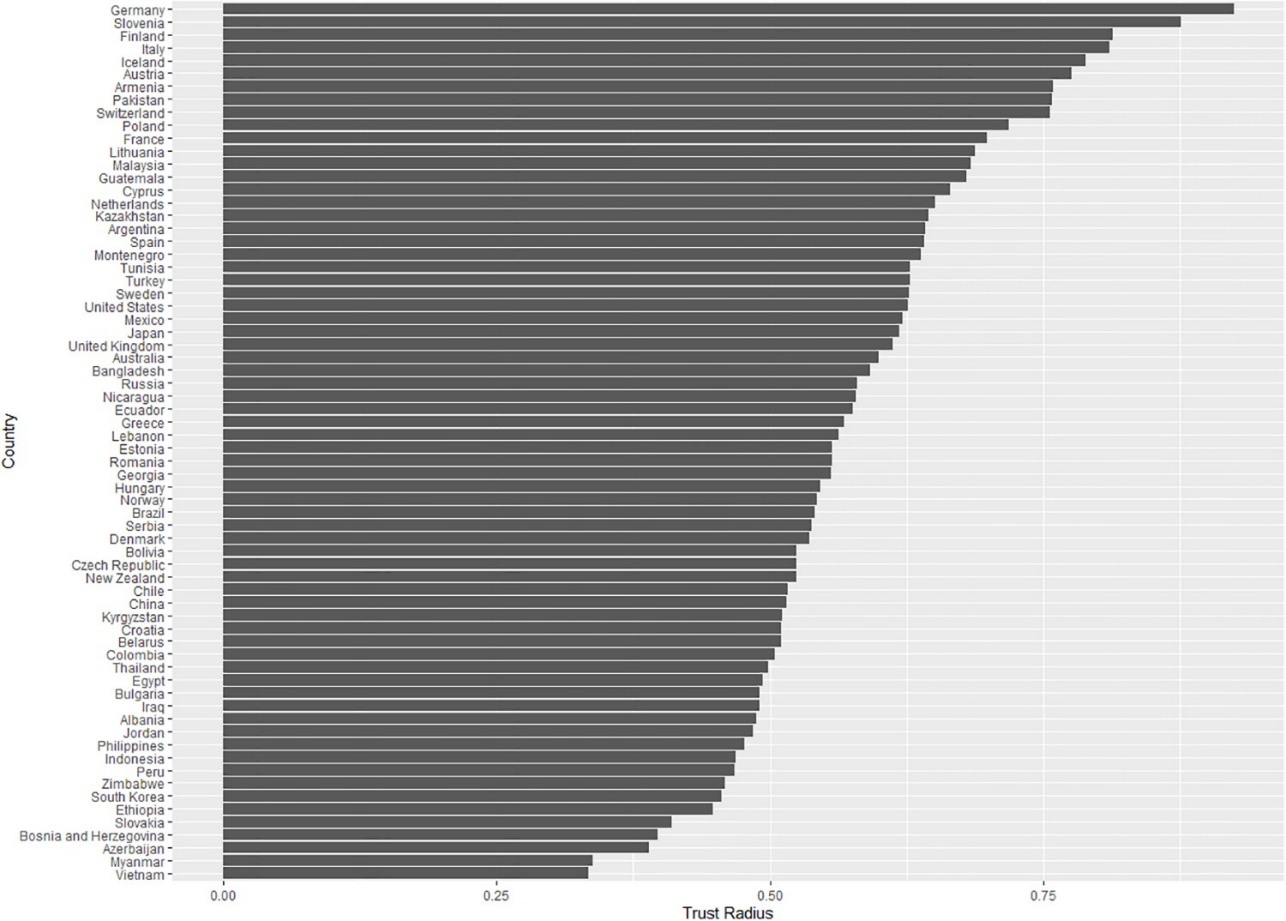
Trust radius across countries.

To define the *trust radius*, the measurements suggested by Delhey et al. [25] were applied in the following three steps. First, unspecified trust was identified. It was defined as a dummy variable coded as 1 when respondents answered the question “Generally speaking, would you say that most people can be trusted or that you need to be very careful in dealing with people? (Code one answer)” by saying “most people can be trusted.” Second, based on the identification, the differences between two associations for each country, namely, associations of unspecified trust with particular and general trust, were calculated. To calculate these differences, the study ran linear regressions by countries where the dependent variable was unspecified trust, and independent variables were the particular and general social trust of respondents. It then identified the coefficients of the independent variables and computed their differences: β_general trust_−β_particular trust_. In line with this definition, the trust radius indicated the extent to which respondents viewed their outgroup people as generally trustworthy. Finally, by adding one to all values, the negative values were fixed. The final measure of the trust radius ranged from zero (the narrowest) to one (the widest). Figs 2 and 3 show variations in the levels of social trust and the trust radius across countries. Some countries like Finland and Switzerland showed high degrees of both social trust and wide trust radius, whereas others such as Peru and Indonesia indicated low degrees.

To disqualify alternative explanations, this study entered multiple control variables as follows. Heterogeneous demographic or economic conditions across countries may create differences in the transmission of COVID-19. Hence, this study controlled the *population* by dividing the number of people by the land area of the country. The *unemployment rate* and log-transformed *GDP* were controlled to reflect the different economic conditions across countries. As countries with high rates of annual overseas movement may have been more vulnerable to the faster transmission of COVID-19, this study used the *international movement expenditure* (% of total imports) and *receipt* (% of total exports) to control for influences from general international movements. These variables refer to the expenditure of international outbound and inbound visitors, respectively. The *death rate* (per thousand) was also included to control the different health conditions of people across countries. Additionally, the number of *hospital beds* (per thousands) is included. This variable controls the differences in the general state of medical treatment across countries because such differences can influence the risk perceptions of people toward a health emergency. All of these data were obtained from the annual World Development Indicators that the World Bank provides in the most recent year [37]. In societies where people are able to communicate using the online space, social distancing may be easier. Thus, this study controlled for the *internet penetration rate* from the Internet World State 2020 by using the percentage of the total population of each country that uses the internet [38].

The faster or slower transmission of COVID-19 can be deeply related to different government interventions. Indeed, as the cases of COVID-19 continue to rise, several countries have entered lockdowns to restrict movement and thus slow down the spread of the virus. These governments’ interventions and the corresponding timing of their implementation may create significant differences in the peaking time for new infections across countries. To control this non-pharmaceutical intervention of each country, this study entered a dummy variable coded as 1 when the focal country was under the *lockdown period* on the focal day, and otherwise coded as 0. The data on the lockdown period for each country were obtained from “National responses to the COVID-19 pandemic” in Wikipedia [39]. Some countries have multiple lockdown periods, depending on their locality. This study used its national standard for lockdown implementation to match the unit of analysis, which is countries.

Finally, the number of *new tests* (per thousand) is an influential element in determining the identification of new infections. However, as several countries (24 of the sample) did not provide this information, missing values were frequently generated. Thus, this study provided results based on models with and without this variable. According to the ECDC, there may be a time gap between the new tests and the announcements of the results owing to unforeseen delays in reporting systems in each country and the time it takes to get the results [34]. To consider this time lag, this study adopted the values at a 5-day time lag that showed a high model fit. This variable was acquired through two data sources: the ECDC and Coronavirus Worldometer [34, 40].

Table 1 shows the descriptive statistics and correlations among the variables used for analysis. Owing to a high correlation (0.83) between particular and general trust, the analyses were conducted separately for both variables. While the death rate is significantly correlated with hospital beds, the maximum value of variance inflation factors was 2.90, indicating no critical multicollinearity problems for the analysis.

**Table 1:** Descriptive statistics and correlations (N = 3,716).

	Variables	Mean	SD	1	2	3	4	5	6	7	8	9	10	11	12	13
1	Reaching the peak	0.02	0.13													
2	Population	157.26	241.00	−0.01												
3	Unemployment rate	5.95	4.02	0.04	−0.09											
4	International movement expenditure	6.74	4.30	0.00	−0.30	0.11										
5	International movement receipts	12.03	12.36	0.01	−0.16	0.06	0.23									
6	Ln (GDP)	26.45	1.73	−0.01	0.01	−0.05	−0.15	−0.39								
7	Death rate	7.96	2.74	0.06	−0.25	0.15	0.06	−0.14	0.00							
8	Hospital beds	3.50	2.88	0.07	−0.16	0.15	0.21	−0.20	0.15	0.69						
9	Internet penetration rate	0.75	0.18	0.06	−0.22	0.22	0.41	−0.25	0.19	0.38	0.58					
10	Lockdown period	0.29	0.45	0.00	−0.06	0.02	0.06	−0.13	0.03	0.13	−0.02	0.06				
11	New tests*	0.24	0.94	0.02	−0.08	−0.03	0.13	0.03	−0.03	−0.02	0.02	0.11	0.05			
12	Trust radius	0.58	0.11	−0.01	0.03	−0.02	0.09	−0.23	0.08	0.04	0.05	0.17	−0.11	0.00		
13	Particular trust	0.72	0.08	0.07	0.25	0.08	0.07	0.12	0.04	0.36	0.38	0.37	−0.28	0.01	0.30	
14	General trust	0.40	0.11	0.06	−0.16	0.05	0.30	0.20	0.05	0.36	0.33	0.52	−0.23	0.07	0.33	0.83

*N = 1,638.

## Results

Table 2 shows the results for the influences of explanatory and control variables on the peaking time for new infections in each country. All variables were standardized for comparison. Models 1 and 2 include only the control variables. According to Model 1, in countries with a higher unemployment rate (β = 0.262, p = 0.028) and those with lower GDP (β = −0.553, p = 0.003), the new infections of COVID-19 sped up to reach the peak faster. As expected, the number of hospital beds also facilitated the fast peaking time (β = 0.351, p = 0.042). These results are consistent with the realities and expected directions. Counterintuitively, the number of total tests did not show significant coefficients.

**Table 2:** Results of regression analyses for social trust.

Variables	Model 1	Model 2	Model 3	Model 4	Model 5	Model 6	Model 7	Model 8
Population	−0.060	−0.009	−0.199	−0.197	−0.014	−0.012	−0.048	0.055
	(0.11)	(0.13)	(0.12)	(0.19)	(0.12)	(0.14)	(0.10)	(0.13)
Unemployment rate	0.262*	0.137	0.231✝	0.188	0.316*	0.245	0.288*	0.266
	(0.12)	(0.16)	(0.13)	(0.17)	(0.13)	(0.19)	(0.12)	(0.16)
International movement expenditure	−0.021	−0.280	−0.037	−0.254	−0.012	−0.273	−0.002	−0.139
	(0.14)	(0.19)	(0.13)	(0.22)	(0.13)	(0.19)	(0.13)	(0.22)
International movement receipts	−0.087	0.178	−0.188	−0.047	−0.174	0.019	−0.084	0.147
	(0.14)	(0.17)	(0.13)	(0.24)	(0.13)	(0.21)	(0.14)	(0.15)
Ln(GDP)	−0.553**	−0.234	−0.637***	−0.307	−0.626***	−0.287	−0.533**	−0.110
	(0.18)	(0.21)	(0.18)	(0.22)	(0.18)	(0.20)	(0.19)	(0.24)
Death rates	−0.034	0.189	−0.211	−0.036	−0.201	0.044	−0.018	0.263
	(0.17)	(0.19)	(0.18)	(0.22)	(0.18)	(0.20)	(0.18)	(0.22)
Hospital beds	0.351*	0.167	0.494**	0.243	0.593**	0.298	0.355*	0.106
	(0.17)	(0.23)	(0.19)	(0.21)	(0.22)	(0.22)	(0.17)	(0.27)
Internet penetration rate	0.137	0.530*	−0.076	0.269	−0.153	0.268	0.116	0.509✝
	(0.21)	(0.26)	(0.18)	(0.30)	(0.24)	(0.31)	(0.20)	(0.26)
Lockdown period	0.139	−0.200	0.300*	−0.059	0.272✝	−0.118	0.170	−0.049
	(0.14)	(0.18)	(0.15)	(0.21)	(0.15)	(0.19)	(0.14)	(0.19)
Trust radius	0.134	0.025	0.084	−0.133	0.044	−0.096	−0.013	−0.327
	(0.15)	(0.25)	(0.15)	(0.26)	(0.16)	(0.27)	(0.15)	(0.24)
New test		−0.019		−0.061		−0.035		−0.040
		(0.09)		(0.16)		(0.12)		(0.09)
Particular trust			0.635**	0.540✝				
			(0.19)	(0.29)				
General trust					0.502*	0.408		
					(0.23)	(0.28)		
(Trust radius)^2^							0.159*	0.444**
							(0.07)	(0.15)
Log pseudolikelihood	−208. 47	−100.33	−201.87	−98.18	−205.40	−99.24	−206.66	−96.62
Wald chi2	30.21***	15.18***	47.78***	27.40**	32.61***	19.01✝	41.36***	31.83**
N	3716	1638	3716	1638	3716	1638	3716	1638

Standard errors in parentheses; All variables standardized for comparisons.

^✝^p < .1;

* p < .05;

** p < .01;

*** p < .001.

Models 3 to 6 represent the results for the influence of particular and general trust. As predicted, in countries with a high level of particular trust, the number of new infections reached a peak faster than other countries (β = 0.635, p = 0.001 in Model 3; β = 0.540, p = 0.065 in Model 4). The influence of general trust has been shown to be weak. It presents a positive and significant coefficient in Model 5 (β = 0.502, p = 0.029) but an insignificant coefficient in Model 6 (β = 0.408, p = 0.150).

**Fig 4:**
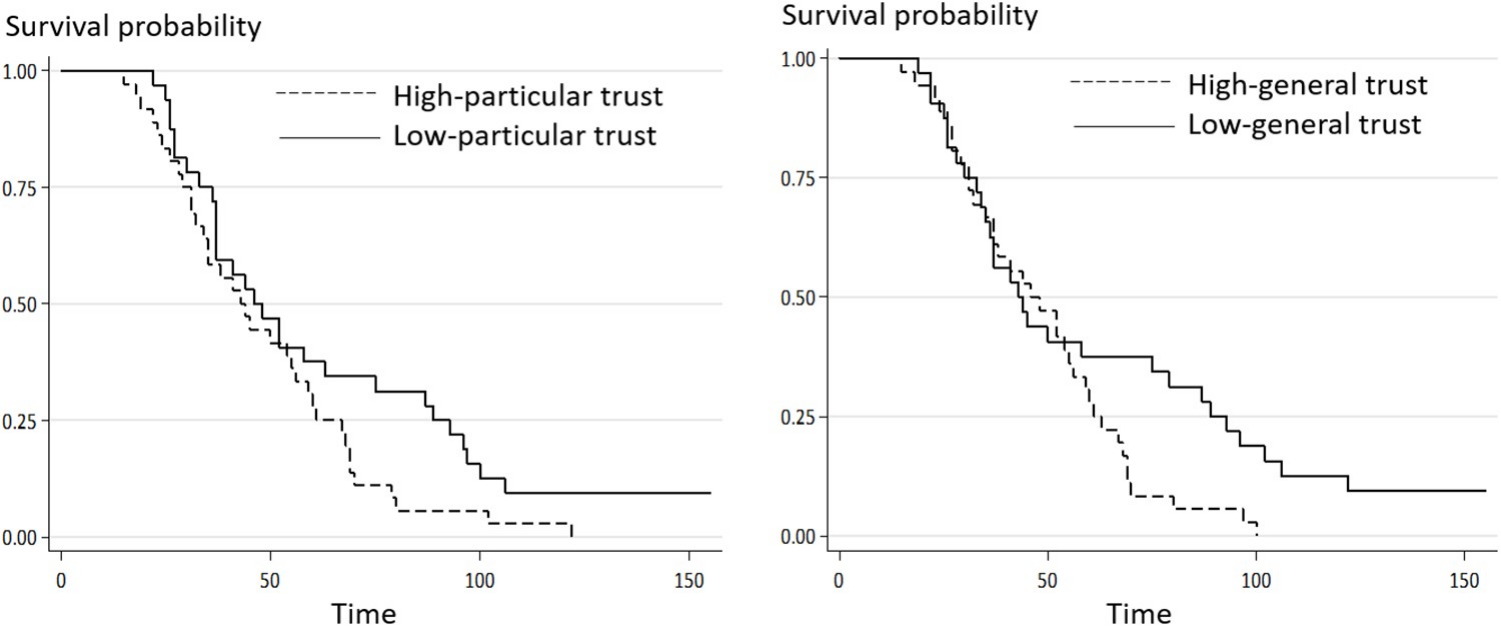
Influence of particular and general trust on the fast peaking time for COVID-19.

To visualize the results, this study separated the sample countries into groups of high and low particular or general trust based on their median values and plotted their survival functions using the Kaplan-Meier estimator. As shown in Fig 4, concerning both particular and general trust, the probabilities of reaching the peak of high- or low-trust countries run parallel over the first 50 days, after which the high-trust countries display a higher hazard of reaching the peak, particularly between 50 and 100 days.

Models 7 and 8 test the curvilinear pattern of the trust radius to explain the time duration that each country would take to reach the peak by including the squared term of the trust radius. The addition of the quadratic terms in Models 7 and 8 leads to significantly better models than do Models 1 (LR chi2 = 3.62, p = 0.057) and 2 (LR chi2 = 7.42, p = 0.006), respectively. The squared terms of the trust radius show a significantly positive impact on the fast peaking time (β = 0.159, p = 0.042 in Model 7; β = 0.444, p = 0.005 in Model 8), suggesting that the fast transmission and control of COVID-19 will be more likely in countries with narrower or wider ranges of trustees than in those with a medium range of trustees. Using the appropriateness test for a U-shaped relationship [41], this study confirms that the given U-shaped relationships are statistically significant (t = 2.00, p = 0.023 for Model 7; t = 2.83, p = 0.002 for Model 8).

To check the robustness of the results, this study redefined the peak only if the given day had the largest number of new infections between 14 days before and after that day, although the total number of cases on that day was less than one for every million. Then, the study reran the aforementioned regressions based on the redefined peak. The results replicated those shown in Table 2, proving that the results are robust with inclusions of the low peaks.

### Additional analysis

To gain additional insight, this study conducted two further examinations. First, it tested how the transmission speed of COVID-19 differed based on the trust level in the public sector, namely, political trust across countries. To define political trust, following previous studies [14], this study used questions in the WVS [15] to understand the extent of trust people vested in their political, governance, and security sector institutions, namely, the parliament, the government, political parties, the justice system, civil services, and the police. The respondents answered these questions by picking from four options: trust completely, somewhat, not very much, and not at all. This study rescaled each answer from zero (no trust) to one (completely trust) and calculated the averages of these answers per country.

The political trust may rely heavily on the county’s political regime, such as the degree of democracy. To control the influence of individual countries’ political regimes, this study included the democracy scores in 2019 for each country as compiled by the Economist Intelligence Unit [42]. This index reflects the different conditions of electoral processes and pluralism, civil liberties, the functioning of the government, political participation, and political culture across countries. Full democracies score high, whereas authoritarian political regimes score low.

Table 3 shows the results. In the same way as that of particular and general trust, high political trust also accelerates the fast peaking time for new infections. These results show consistency with previous arguments concerning the importance of trust in the public sector in the promotion of expected health behaviors of people and facilitation of their compliance with health rules set by public sectors [16]. However, it is also noteworthy that these results imply the possibility of faster transmissions during the early phase of pandemic due to high optimism in societies with high political trust.

**Table 3:** Results of regression analyses for political trust.

Variables	Model 1	Model 2
Population	−0.263✝	−0.214
	(0.14)	(0.16)
Unemployment rate	0.435**	0.261
	(0.14)	(0.17)
International movement expenditure	0.083	−0.195
	(0.13)	(0.23)
International movement receipts	−0.204	0.067
	(0.16)	(0.20)
Ln(GDP)	−0.752***	−0.397✝
	(0.18)	(0.22)
Death rates	−0.177	0.075
	(0.18)	(0.20)
Hospital beds	0.619**	0.318
	(0.20)	(0.21)
Internet penetration rate	−0.325	0.332
	(0.24)	(0.28)
Lockdown period	0.261✝	−0.024
	(0.15)	(0.21)
Trust radius	0.114	−0.034
	(0.15)	(0.22)
Democracy scores	0.351✝	0.083
	(0.19)	(0.19)
New test		−0.135
		(0.27)
Political trust	0.677***	0.626**
	(0.19)	(0.23)
Log pseudolikelihood	−201.22	−97.00
Wald chi2	53.51***	35.65***
N	3716	1638

Standard errors in parentheses; All variables standardized for comparisons.

^✝^p < .1;

* p < .05;

** p < .01;

*** p < .001.

Second, this study argues that the fast peaking time for new infections in high-trust societies results from people’s perception of low risk towards an emergency and strong compliance with social rules in responding to common adversities in those society. To gain further insights concerning this argument, this study conducted an analysis to test how social trust affects the mobility of people in a pandemic. If the argument holds true, the rate of mobility should be generally high in high-trust countries; however, the high mobility in high-trust countries should be effectively controlled through stringent restrictions such as lockdowns.

To test this prediction, this study utilized the data on Community Mobility Reports provided by Google [43]. This data show the movement trends of people around the world, beginning on February 15 until the latest date on record (November 8), across six categories of places: retail and recreation, groceries and pharmacies, parks, transit stations, workplaces, and residential areas. The data describe changes in visits and length of stay in these places compared to a baseline measured using the median value during the five-week period from January 3 to February 6, 2020. Since the values in the six categories show high correlations with each other, this study focused only on the data on mobility in transit stations for analysis.

In the analysis models, this study controlled the population and log-transformed GDP to consider the countries’ different conditions. The internet penetration rate was also controlled to reflect the availability of communication through the online space. Since mobility will be influenced by the severity of COVID-19 transmissions, this study entered the number of new infections per million people on the focal day in each country. Finally, the lockdown period is also controlled to reflect the influence of government interventions on mobility. For analysis, generalized estimating equations (GEE) with robust standard errors were applied. GEE models are appropriate for testing panel datasets since they are effective for controlling autocorrelation and heteroskedasticity. An identity link and a Gaussian distribution were applied for the tests.

**Fig 5:**
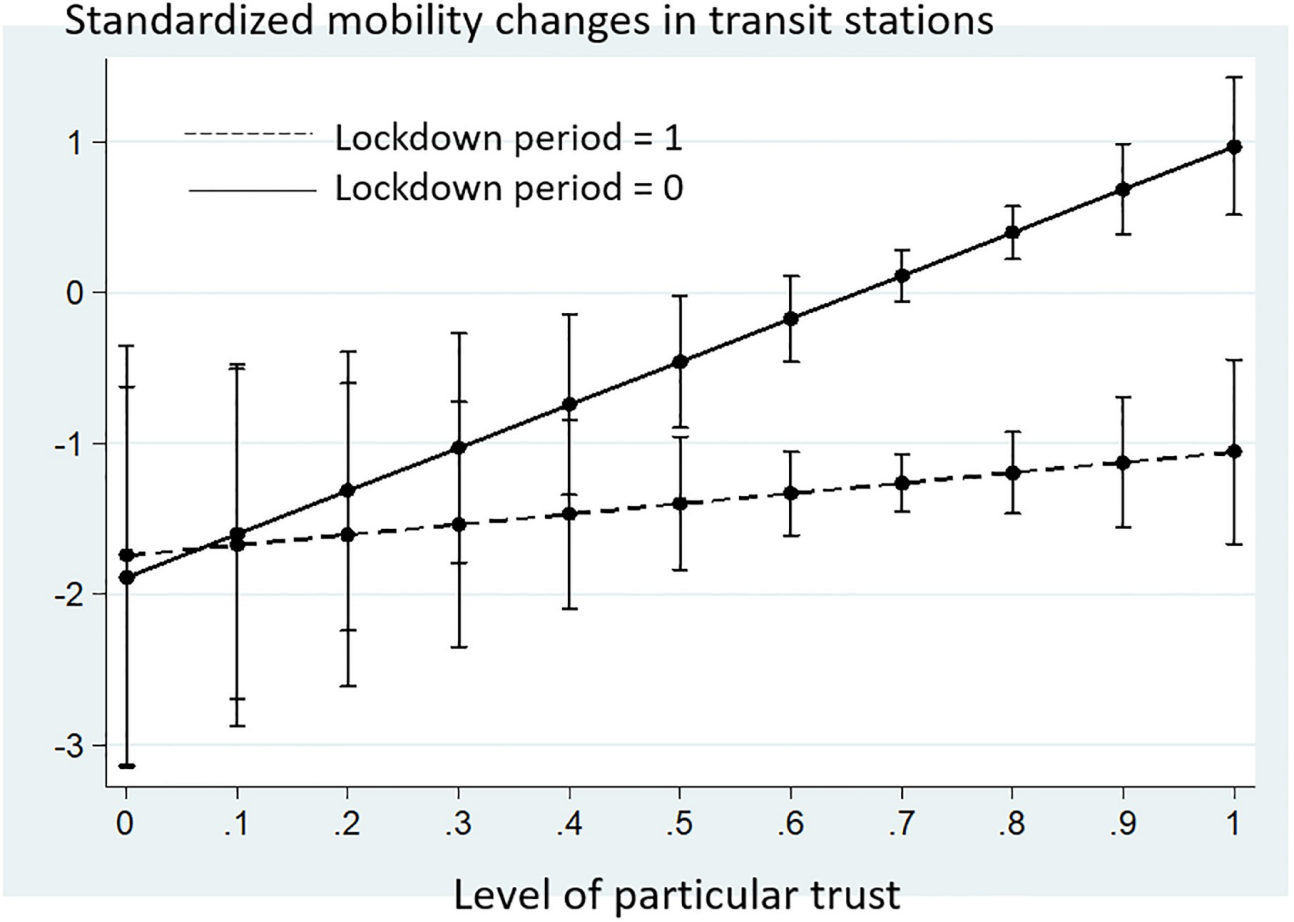
Interaction plot for particular trust and lockdown period.

Table 4 shows the analysis results. All the variables are standardized. According to Model 1, the number of new infections decreases the mobility of people (β = −0.026, p = 0.087). As expected, mobility was intensely regulated during the lockdown period (β = −0.517, p<0.001). Model 2 shows that in countries with strong ingroup trust (high-particular trust), mobility tends to be significantly high under the pandemic (β = 0.198, p = 0.001). Contrary to this, Model 3 shows that general trust does not significantly change mobility rates. Model 4 presents that the influence of particular trust was significantly alleviated during the lockdown period of countries (β = −0.063, p = 0.010). Fig 5 illustrates this tendency. In this figure, the dotted and bold lines indicate the influence of particular trust on the mobility of people in transit stations under the lockdown period and during regular periods, respectively. Fig 5 shows that particular trust of countries is positively related to high mobility of people during the pandemic, but this tendency is significantly weakened during lockdown period. All of these results show consistency with the arguments that are presented in this study.

**Table 4:** Results of regression analyses for mobility changes.

	Model 1	Model 2	Model 3	Model 4	Model 5
Population	−0.089	−0.117	−0.085	−0.113	−0.086
	(0.08)	(0.07)	(0.08)	(0.07)	(0.08)
Ln(GDP)	0.132	0.151	0.133	0.155	0.140
	(0.10)	(0.10)	(0.10)	(0.10)	(0.10)
Internet penetration rate	0.070	−0.022	0.024	−0.026	0.021
	(0.08)	(0.08)	(0.11)	(0.08)	(0.11)
Number of new infections	−0.026✝	−0.026✝	−0.026✝	−0.022	−0.025
	(0.02)	(0.02)	(0.02)	(0.02)	(0.02)
Lockdown period	−0.517***	−0.517***	−0.517***	−0.528***	−0.520***
	(0.04)	(0.04)	(0.04)	(0.04)	(0.04)
Trust radius	0.015	−0.007	0.006	−0.006	0.006
	(0.06)	(0.06)	(0.06)	(0.06)	(0.06)
Particular trust		0.198**		0.200**	
		(0.06)		(0.07)	
General trust			0.074		0.074
			(0.10)		(0.10)
Particular trust ×				−0.063*	
Lockdown period				(0.02)	
General trust ×					−0.028
Lockdown period					(0.04)
Constant	−0.005	−0.004	−0.005	−0.010	−0.005
	(0.08)	(0.07)	(0.08)	(0.07)	(0.08)
Wald chi2	330.61***	358.88***	381.71***	374.28***	414.82***
N	15335	15335	15335	15335	15335

Standard errors in parentheses; All variables standardized for comparisons.

^✝^p < .1;

* p < .05;

** p < .01;

*** p < .001.

## Discussion

This study examined the influence of social trust on the spread of COVID-19 across 68 countries between December 31, 2019 and July 31, 2020. According to the results, in countries characterized by high levels of social trust (especially the particular social trust) or a narrower or wider range of trustees, the number of new cases is likely to reach the peak faster than in other countries. These findings have the following implications for practice and the literature.

First, social trust brings about the current good-news-bad-news conundrum. On the one hand, social trust may help speed up the neutralization of COVID-19 after the spread reaches a certain level because social trust facilitates cooperation among people in solving their common adversities as well as compliance to social rules for health-promoting behaviors. This observation is in parallel with other previous arguments that emphasize the benefits of social trust in managing COVID-19 transmissions [12, 13]. Nevertheless, on the other hand, before such neutralization takes place, social trust may also enable the faster transmission of COVID-19 because in high-trust societies, people tend to be optimistic toward risks and engage in collective behaviors based on face-to-face interactions, particularly in times of crises. They may strengthen communal behaviors in the same religious groups and may evince greater concern for their family members or neighborhoods by frequently contacting them. This would be a natural phenomenon in high-trust societies because dense and cohesive social relations constitute an important mechanism that enables people to overcome adversities they may have encountered [44, 45]. In connection, the requirements for social distance or a high-risk perception in such societies can be somewhat paradoxical and challenging. This highlights that an equal emphasis on the risks of social trust will be needed along with its benefits in order to cope with the present crisis, particularly in the early phase in pandemics.

Second, the results of this study call for further studies on risks associated with social trust. Although it is apparent that social trust generates numerous forms of social capital, as several previous studies have shown [1, 5, 46], it also generates unexpected problems, as the present study has suggested. For a more balanced development of the literature on the effects of social trust, we need more empirical research on the other spectrum of social trust that previous studies have neglected. In line with this argument, although some previous studies have suggested the potential harm of high trust among social actors in innovation [47], team management [48], and entrepreneurship [49], there may be additional critical side effects about which we have yet to learn.

Addressing a balanced discussion on the effects of social trust will be critical for theoretical development and for comprehending social implications. As shown in this study, social trust is an intense and stable mechanism that explains actors’ social behaviors. According to the results, in high-trust societies, in-person contact, particularly contact among ingroup members, may be less controllable even in an emergency in which social distance must be maintained. This tendency does not change while controlling the internet penetration rate in society. This suggests that even in environments in which people can maintain contact online, routinized in-person or intimate contact within groups may be less controllable.

In this line of arguments, other relevant factors such as group or national identity might also show similar effects as that of social trust. For instance, Van Bavel et al. [50], in their recent research, have suggested that a sense of strong group or national identity will play a critical role in motivating people to invest efforts that combat the pandemic. This is because such strong group identity facilitates mutual cooperation and adherence to norms to protect the group’s welfare. The results of this study also support these arguments with regard to the significant and strong influence of the particular trust in societies. However, the results also indicate that the cohesive societies such as those with strong ingroup trust or identity might also see speedier transmission of COVID-19 before neutralization takes place. This is because the members of such societies are likely to be involved in intimate interactions within the group and may have a strong sense of security from their societies before they recognize risks.

Lastly, according to the results of this study, societies may need to implement different strategies to respond to pandemics on the basis of their levels of social trust. For instance, in high-trust societies, the early phase of the pandemic will be a critical duration for controlling the crisis. With the strong tendency for compliance to social norms or rules in those societies, people will be significantly influenced by what others are doing and which rules are imposed upon them. Hence, strong restrictions against close contact and the dissemination of information through social media or networks about behavioral standards should be given at the early phase of the crisis, so that the societies can form the norms and rules for health behaviors immediately. In the low-trust societies, on the other hand, the policies and strategies should take longer-term perspectives because it may take more time to start neutralizing the pandemic.

This study has several limitations. First, the varying duration of the peaking time for COVID-19 across countries will depend largely on restrictions imposed by government policies, and particularly on the strength and timing for the adoption of restrictions. For this reason, this study included a control variable that measures the lockdown periods of each country in the analysis. However, due to limited access to data, the study could not fully take into account many other potential influences from different government policies in the analysis. For more political implications, future studies will benefit from testing how the effects of government restrictions interact with the different levels or radius of social trust in countries when it comes to managing a global pandemic.

Second, the discussion did not cover how long the countries take to revert to the normal state and how social trust may affect the second and third waves of infections following the first wave. As societies are still in the course of COVID-19 transmission, it is premature to examine these aspects at this point. Follow-up studies in the future should strive to deepen our understanding of the relationship between social trust and epidemic transmission.

Third, this study uses each country as a unit of analysis, and the results thus did not reflect the influences of individual differences among the respondents. This is a critical limitation because compliance to social rules is significantly affected by individual aspects. Using alternative methods of analysis such as surveys and experiments at the individual level, future studies can provide more robust results.

Finally, although this study, to a limited degree, focused on social trust as an antecedent of the fast peaking time for the transmission of COVID-19, many other socio-behavioral factors, such as the national culture, ethnic or religious diversities, and time perspective of the societies, may affect the peaking time through behavioral control and restriction among people. Future research that compares the influences of these diverse factors across countries will suggest further useful strategies to manage this global crisis.

Despite these limitations, the theoretical arguments and empirical findings of this study offer important implications for the literature on social trust as well as the practical control of the current global crisis. It is hoped that the approach and findings of this study contribute to holistic safety in society.

## Supporting information

S1 File Dataset for main analysis. (DTA)

S2 File Dataset for mobility analysis. (DTA)
